# Epigenomic and Metabolic Interplay in the Development of Metastatic Brain Tumors

**DOI:** 10.32604/or.2026.072620

**Published:** 2026-02-24

**Authors:** Vishal Rastogi, Deepak Verma, Saurabh Verma, Prakash Haloi, Shruti Kapoor, Havagiray R. Chitme, Nethaji Muniraj, Priyanka Saroj

**Affiliations:** 1 Department of Pharmacology, Amity Institute of Pharmacy, Amity University, Noida, 201313, India; 2 Department of Oncology, Johns Hopkins University, Baltimore, MD 21210, USA; 3 Department of Pharmaceutics, Amity Institute of Pharmacy, Amity University, Noida, 201313, India; 4 Molecular, Cellular and Developmental Biology, University of California Santa Cruz, Santa Cruz, CA 95064, USA; 5 Center for Cancer and Immunology Research, Children’s National Hospital, 111 Michigan Ave NW, Washington, DC 20010, USA

**Keywords:** Metabolic reprogramming, brain tumor, epigenetic alteration

## Abstract

Metastatic brain tumors undergo profound metabolic–epigenetic reprogramming driven by the unique constraints of the brain microenvironment. Hypoxia-inducible factor-1α (HIF1α) enhances glycolytic flux, lactate accumulation, and histone lactylation, collectively supporting metastatic colonization and immune evasion. Key metabolites including acetyl-CoA, S-adenosylmethionine (SAM), α-ketoglutarate (α-KG), fumarate, and 2-hydroxyglutarate (2-HG)—directly modify chromatin states by regulating histone acetyltransferases, DNA/histone methyltransferases, and α-KG dependent dioxygenases such as Ten-Eleven Translocation (TET) enzymes and lysine demethylases (KDMs). These metabolic shifts result in aberrant DNA methylation, histone lysine residue at position 27 on Histone H3 (H3K27) trimethylation, and depletion of 5-hydroxymethylcytosine (5hmC), all of which are hallmark epigenetic alterations in brain metastasis and primary Central Nervous System (CNS) tumors. Additionally, the blood–brain barrier (BBB) and blood–tumor barrier (BTB) impose nutrient restrictions and induce metabolic dependency on glutamine, acetate, and lactate shuttling, thereby reshaping epigenetic enzyme activity. We synthesize current mechanistic evidence showing how metabolic pressures in the brain microenvironment remodel the epigenome to promote tumor plasticity, stemness, and therapeutic resistance. Understanding these coupled pathways reveals vulnerable nodes such as HIF1α signaling, α-KG–dependent demethylation, and lactate-driven epigenetic remodeling that may be exploited for targeted treatment of metastatic brain tumors. The present review aims to provide in-depth insights into epigenetic regulation, including chromatin and histone modifications as well as noncoding RNAs and metabolic reprogramming, highlighting how the two interplay in the development and progression of metastatic brain tumors and their therapeutic potential.

## 1 Introduction

Blood-brain barrier invasion is associated with brain metastasis [1]. Metastatic brain tumors represent one of the most complex and lethal manifestations of systemic cancer progression. Most adult cancer patients develop brain metastases within ten to twenty years of their onset [2]. Colonizing the brain decreases the metabolic flexibility of cancer cells. Metastasis and survival are also supported by initial metabolic plasticity [3]. There is still a mystery about how primary cancer cells can cross the blood–brain barrier to enter brain tissues despite improved management of brain metastasis [4]. Despite advances in oncologic therapies, brain metastases remain associated with poor prognosis, largely because metastatic cancer cells undergo extensive molecular adaptation to survive the distinctive biochemical, metabolic, and immunological conditions of the brain. Epigenomics and metabolism play a crucial role in these adaptations. Metastatic brain tumors are driven by epigenetic and metabolic processes that interact dynamically [5]. Along with metastatic spread to the brain, epigenetic alterations and metabolic adaptations promote tumor growth and development [6]. The fate and nature of cancerous cells are dramatically altered by epigenetic changes without altering the DNA sequence. These modifications regulate the expression of oncogenes or tumor suppressor genes, which can either be activated or suppressed, thereby promoting cancer progression and metastasis [7,8]. Metastatic brain tumors undergo metabolic reprogramming, resulting in their aggressive behavior and resistance to therapeutics [9]. The tricarboxylic acid (TCA) cycle and altered energy metabolism are crucial metabolic pathways to sustain tumor growth [10]. Metabolic reprogramming not only drives tumor cell proliferation but also provides energy and redox requirements necessary for sustaining malignant growth [11]. Simultaneously, the relationship between metastatic tumor cells and the brain microenvironment is also crucial for the growth and spread of cancer cells in the brain [12]. Growing evidence in the field suggests that understanding the coordinated interplay of epigenetic modifications and metabolism in developing brain tumors is essential for identifying new therapeutic avenues for patients with these conditions. By disrupting the supportive interplay between cancer cells’ metabolism and epigenetic modifications, researchers aim to discover more effective treatment strategies for patients with metastatic brain tumors [13,14].

Furthermore, the interaction between circulating tumor cells and endothelial cells has been found to be mediated by adhesion molecules [15]. Blood-brain barrier (BBB) limits the entry of nutrients such as glucose and amino acids, creating a specific metabolic challenge for metastatic cells that leads to epigenetic adaptations [16]. The transmigration efficiency of BBB cells is less than 0.05%, making them more challenging to cross. As a result, endothelial barrier function varies between organs [17]. Apoptosis, cell motility, invasion, and angiogenesis are all regulated by α2integrin on tumor cells [18]. Tumor-derived extracellular vesicles may indeed cause endothelial cells to undergo apoptosis. Invasion of the brain by circulating tumor cells may also be triggered by endothelial apoptosis (Reymond et al., 2013). It is possible to have fenestration and extravasation of endothelial cells following transcytosis, which may contribute to the fenestration of tumor cells [19]. In addition to decreasing endothelial permeability, hyperhomocysteinemia lowers oxidative stress. Exosomes derived from the BBB may help treat BBB disorders. By paracrine signaling, astrocytes produce cytokines that increase brain metastatic tumor growth. Unlike all other metastatic sites, the brain is more conducive to tumor growth once tumor cells enter [20,21]. Normal brain function depends on endothelium, pericytes, and astrocytes. The blood-tumor barrier (BTB) develops during tumor growth and exhibits heterogeneous leakiness, allowing varied delivery of metabolites and drugs, which influences epigenetic enzyme activity [22]. BTB is formed when the BBB is disrupted during tumor progression. Astrocytic endfeet and neuronal connections are lost in the BTB, which is more permeable than the BBB [23]. Physical dislocation of astrocytic endfeet is associated with glioma cell invasion [24]. According to T-cell subpopulations and peripheral monocytes, brain tumors are highly permeable to circulating immune cells [25]. Brain metastases (intratumoral vasculature) fail to reestablish a normal BBB due to decreased junctional proteins in BTB [26]. A tumor cell’s size is limited in the capillary bed upon entering the brain vasculature. Enters the brain parenchyma after extravasating across the BBB. HBEGF, COX2, and ST6GALNAC5 mediate cancer cell migration across the BBB [27]. It has also been proposed that integrin (αvβ3 and β1) activation controls tumor-cell arrest and adhesion to vessels [28–30]. Metastatic tumors can spread into their hosts by invading stroma and escaping the immune system [31] through perivascular expansion of extravascular-tumour cells, and obtain adequate nutrients for their proliferation through new blood vessels (angiogenesis) [32]. The metastatic cascade begins with tissue invasion followed by intragastric embolism, which involves entanglement in narrow capillaries due to tumor cell proliferation. Cancer cells may remain dormant in the surrounding tissue for an indeterminate amount of time before migrating out of the blood vessel (extravasation), forming metastatic lesions after proliferating. Thus, the ‘seed and soil’ hypothesis, first proposed by Paget in 1889, holds that metastatic disease does not occur at random, but is determined by compatibility between the origin tumor and the target organ or tissue [33].

As ATP production is boosted to overcome energy deficits, metastatic cells prioritise survival over proliferation [5]. Metastatic brain tumors are associated with changes in metabolism and epigenetics in their microenvironment by reprogramming cellular metabolism, and oncogenic mutations drive tumorigenesis [34]. Metabolism reprogramming is crucial for metastatic tumor development, as it allows cells to switch between epithelial and mesenchymal states [9]. Furthermore, metastatic cancer cells dynamically and selectively adjust their metabolism throughout the metastatic cascade [35]. Cancer stem cells (CSCs) are reprogrammed by aberrant epigenetic machinery, which preserves their characteristics in the long term [36]. In addition to affecting surrounding cell phenotypes, epigenetic modifications contribute to favorable tumor microenvironments [37]. A wide range of pediatric brain tumors are driven by epigenetic dysregulation [38]. Epigenetic modifications, specifically DNA methylation and miRNA molecules, strongly influence tumor development and occurrence [39]. A tissue-based epigenetic classification as well as therapeutic subtypes have been developed to treat metastatic brain tumors [40]. Epigenetic mechanisms are critical for understanding neuro-oncology, as well as tumors originating from the CNS [41]. Epigenetic changes and covalent histone modifications are common in pediatric brain tumors [42]. The analysis of molecular data from brain tumors concluded that epigenetic alterations have significant implications for glioblastomas [43]. Cancer cells are metabolically reprogrammed by tumor suppression of LKB1, which drives energy production and provides biosynthetic intermediates for growth [44].

Thus, this review aimed to provide strong insights into epigenetic regulation, metabolic reprogramming and the crosstalk between the two, leading to initiation, progression, and shaping the microenvironment of metastatic brain tumors and leveraging them as prospective therapeutic potential.

## 2 Alterations in Molecular Epigenetics

Cancer is increasingly believed to be a metabolic disease. In metabolic regulation and tumorigenesis, epigenetic regulation plays an important role [45]. It has been proven that genetic and epigenetic changes can affect tumor cell plasticity and changes in the rewiring of cell signals, which in turn can affect cancer cell survival, growth, and metastasis. Many diseases, particularly brain tumors, are profoundly influenced by metabolic environments and epigenetic modifications [45]. There is significant potential for epigenetic research in brain metastases.

### 2.1 Epigenetic Remodeling during Brain Metastases

#### 2.1.1 DNA Methylation

**Figure 1:**
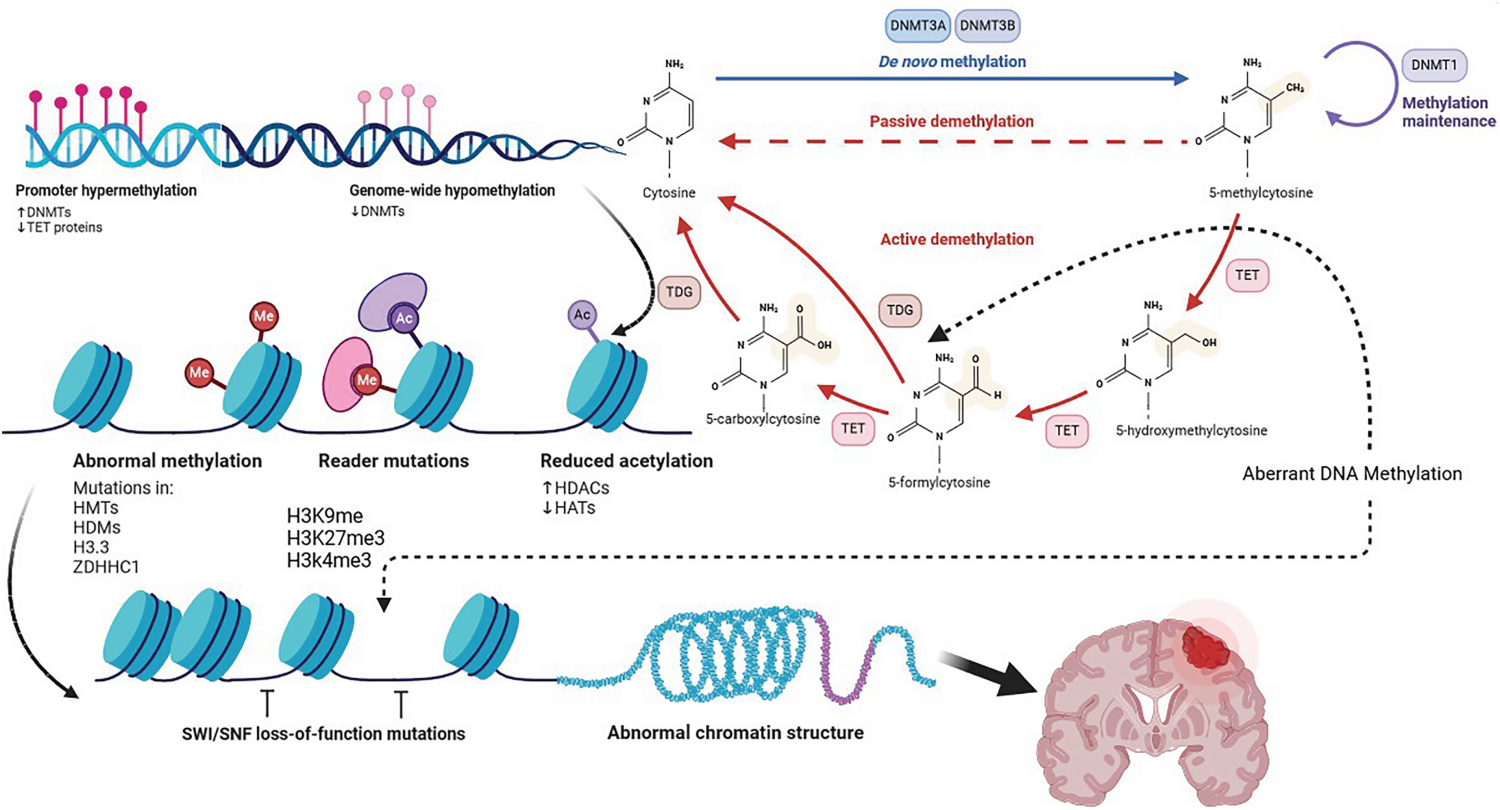
DNA Methylation, Created with BioRender.com. As a result of the conversion of S-Adenosyl-L-Methionine (SAM) to S-Adenosyl-L-Homocysteine (SAH), DNMTs catalyze the methylation of 5-methylcytosine from cytosine. Cytosine-phosphate-Guanine dinucleotide (CpG) island hypermethylation in promoter regions suppress transcriptional gene activation. An enzyme called TET activates transcriptional genes by hydroxylating 5mC. DNMT1, DNMT3A, and DNMT3B also contribute to that maintenance and augmentation. As cofactors, acetyl-CoA and SAM are used by the writers of acetylated and methylated marks. Using erasers such as HDACs and KDMs, one can remove acetylated and methylated marks. The red-dashed line shows passive demethylation, the red line shows active demethylation, the blue line shows *de novo* methylation, and the black-dashed line shows aberrant DNA methylation. SAH means S-adenosylhomocysteine; SAM means S-adenosylmethionine; TETs means Ten-eleven translocation family. HATs represent histone acetyltransferases, HDACs represent histone deacetylases, KDMs represent histone demethylases, and DNMTs represent DNA methyltransferases. Abb: DNA Methyltransferase (DNMT), Ten-Eleven Translocation (TET), Thymine DNA Glycosylase (TDG), Histone Deacetylase (HDAC), Histone Acetyltransferases (HATs), Histone Methyltransferase (HMT), Histone Demethylase (HDM), Zinc Finger DHHC-Type Palmitoyltransferase 1 (ZDHHC1), Histone H3 Variant 3 (H3.3), Histone H3 Lysine Residue (H3K)

A methyl group is added to cytosine during DNA methylation, thereby producing 5-5-methylcytosine. DNA methyltransferases (DNMTs) control DNA methylation by creating or maintaining methylation patterns [46]. A more detailed depiction of DNA methylation mechanisms is shown in Fig. 1. Cancer development and tumorigenesis are mainly due to abnormal methylation in tumor suppressor genes. The DNA methylation control mechanism is disrupted in many diseases, including cancer. CpG islands, which influence gene transcription, play a leading role in regulating these patterns. Cancer cells have aberrant DNA methylation patterns (mutations within Partially Methylated Domains (PMDs), hypomethylation within PMDs, and site-specific hypermethylation) [47]. Histone modifications at DNA methylation sites determine the localization and activation of DNA methyltransferases when they are generally inactive. Consequently, aberrant DNA methylation can trigger cellular oncogenesis by silencing tumor suppressor genes (TSGs). Early findings found that several TSGs are hypermethylated in cancer [47]. ZDHHC1, via zinc finger DHHC-containing 1, is abnormally hypermethylated in various cancers, suppressing glucose metabolism and PPP [48]. Increased DNA methylation is also evidence of epigenetic switching in DNA methylation valleys (DMVs) marked by constitutive heterochromatin [49]. Late-replicating chromatin domains may be associated with demethylated CpG island regions in Glioma CpG islands. Malignant progression may be caused or exacerbated by demethylation. Several growth-related genes will be overexpressed, and tumor growth will be accelerated by DNA demethylation at promoter CpG islands. As chromatin replicates, late replication regions are more likely to be demethylated. DNMT1 maintains DNA methylation by detecting hemimethylated sites and replicating their patterns [50]. When this enzyme is not functioning correctly, the methylation of cells gradually decreases. Demethylation of DNA occurs more frequently in tumors that exhibit higher levels of cell cycle- related genes and occurs more frequently in late-replicating, nuclear lamina-associated domains of cancers [51].

Human and mouse genomes exhibit several features that will influence DNA methylation levels:

1. CpG dinucleotide sequence and replication timing.
2. Number of cell divisions accumulated and presence of Histone H3 Lysine 36 Trimethylation (H3K36me3) histone mark.
3. In PMDs, CpG density and WCGW sequence play an important role in DNA methylation loss per CpG, which is maintained and augmented by DNMT1 and DNMT3A and DNMT3B, respectively [50,52].

IDH-mutated and IDH-wildtype samples (isocitrate dehydrogenase gene) were compared to determine glioma CpG island methylator phenotype.

Mutant IDH1/2 enzymes convert α-ketoglutarate (α-KG) into the oncometabolite D-2-hydroxyglutarate (2-HG), which then competitively inhibits α-KG–dependent dioxygenases, such as TET family DNA demethylases and JmjC-domain histone demethylases [53]. This inhibition leads to extensive DNA and histone hypermethylation, resulting in the characteristic G-CIMP (glioma CpG island methylator phenotype) first identified in diffuse gliomas [54].

In addition to reduced representation bisulfite sequencing profiles and DNA methylation differences in the tumor microenvironment, significant differences in immune cell infiltration were observed for the three transcriptional subtypes. Several studies linked recurring tumors with CD68- and CD163-positive cells, and shorter PFS was linked with fewer pro-inflammatory immune cells and fewer MIB-1-positive, proliferating cells [55]. Glioblastoma epigenome deregulation is mediated by EZH2, as evidenced by the enrichment of EZH2-binding sites among loci. Infiltration of immune cells, the extent of necrosis, and the shape of nuclei of tumor cells were all predicted by DNA methylation. Glioblastoma tumors characterized by the worst survival showed increased EZH2-binding activity after DNA methylation depletion at regulatory elements [56,57]. Ten-eleven translocation proteins (TET) generate 5 hmC from 5 mC during DNA demethylation [58]. CDKNA methylation increases SETDB1 expression, resulting in the uncontrolled growth of tumor cells [59]. De Souza et al. showed that IDH-mutant gliomas exhibit a unique DNA methylation pattern, the glioma CpG island methylator phenotype (G-CIMP), which is strongly linked to epigenetic programming of the oligodendrocyte lineage [60]. This pattern indicates a developmental path where tumor cells keep features of oligodendrocyte progenitors, affecting gene expression and tumor behavior. G-CIMP presence correlates with a better prognosis and less aggressive tumours, highlighting that lineage-specific epigenetic states influence glioma biology and can serve as a basis for molecular classification and targeted therapy. Fig. 1 provides a more detailed overview of the mechanisms underlying DNA methylation. Several key DNA methylation pathways and their underlying mechanisms are summarized in Table 1, highlighting their involvement in histone modification.

**Table 1:** Involvement of DNA methylation pathways and mechanisms modification of histone

The pathways	Involved mechanisms
Methylation of MGMT promoters [61]	Promoter methylation
G-CIMP Phenotype and IDH Mutations [62]	Promoters methylated. In glioma, the CIMP is characterised by hypermethylation. Produce-2-hydroxyglutarate. Inhibit DNA demethylases
PRC2 **(**Polycomb Repressive Complexes 2**)** Targeting [63]	H3K27me3. By methylating histones and interacting with DNA methylation pathways, EZH2 inhibits tumor suppressor gene expression.
Regulation of microRNAs [64]	DNMT targeting. Tumor epigenetic dysregulation has been linked to altered miRNA expression.
Levels of the 5-hmC enzyme and the Ten-Eleven translocation enzyme [65]	5-hmC is formed when 5-mC is oxidized. Resulting in aberrant DNA methylation patterns or reduced 5-hmC levels, also decreased enzyme activity of TET

Note: Abb: O6-Methylguanine-DNA Methyltransferase (MGMT), Isocitrate Dehydrogenase (IDH1/IDH2), Glioma CpG Island Methylator Phenotype (G-CIMP), Trimethylation of lysine 27 on histone H3 (H3K27me3), Enhancer of Zeste Homolog 2 (EZH2), DNA Methyltransferase (DNMT1, DNMT3A, DNMT3B), Ten-Eleven Translocation dioxygenase family (TET1, TET2, TET3), 5-Hydroxymethylcytosine (5-hmC), 5-Methylcytosine (5-mC).

#### 2.1.2 Histone Modification

**Figure 2:**
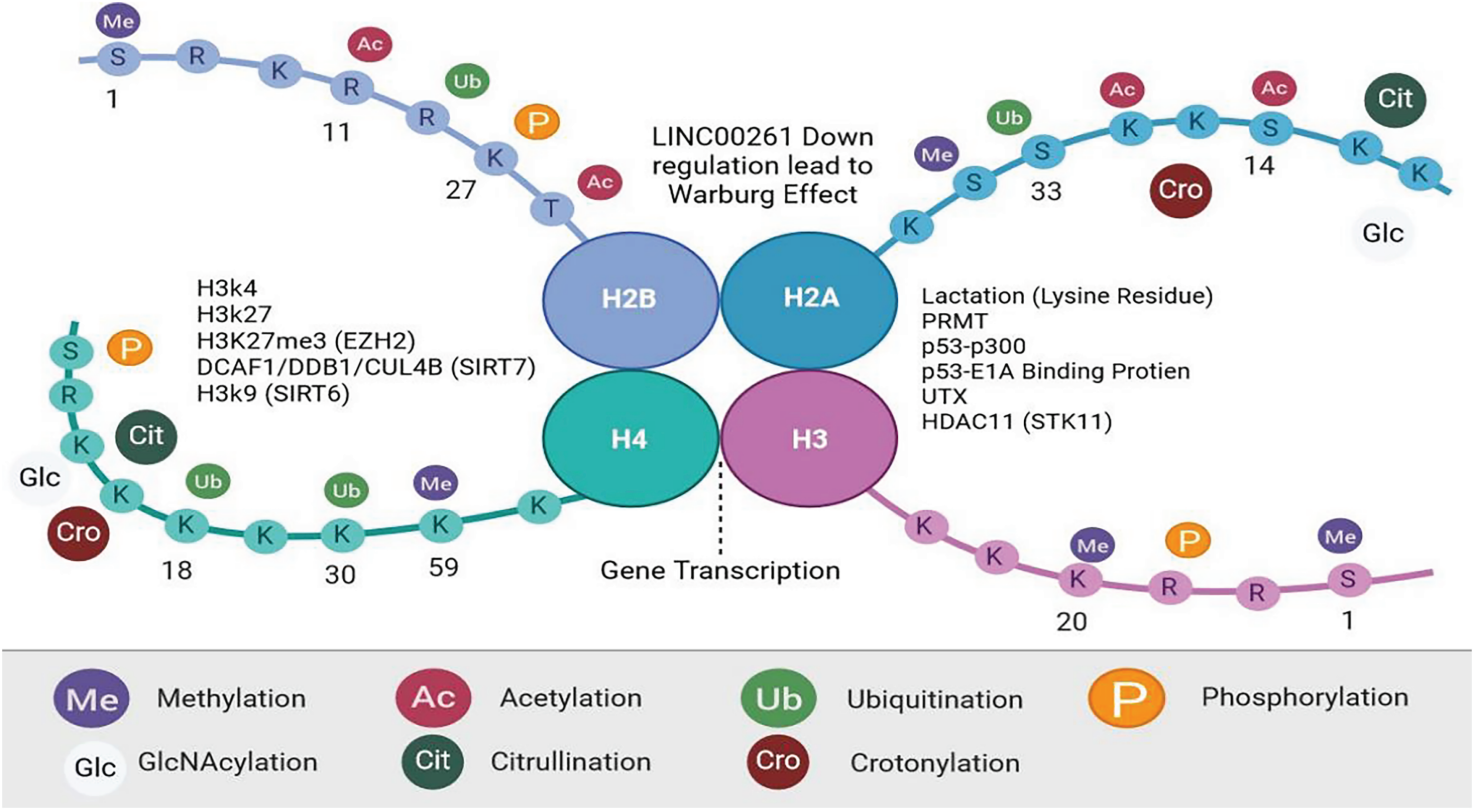
Histone Modification, Created with BioRender.com. During posttranslational modification (PTMs), histones H2A, H2B, H3, and H4 are altered. In addition to methylation, acetylation, ubiquitination, and phosphorylation, there are four major (PTMs). Crotonylation, GlcNAcylation, and citrullination are among the histone modifications identified. Histone proteins contain amino acids at their N- and C-terminus, along with globules within their nucleosome cores that function as histone-modifying enzymes. Abb: Protein Arginine Methyltransferase (PRMT), Serine/Threonine Kinase 11 (STK11), sirtuin (SIRT), Enhancer of Zeste Homolog (EZH), Ubiquitously Transcribed Tetratricopeptide Repeat, X-linked (UTX)

H3 and H4 nucleosome tails undergo post-transcriptional modifications at their N-terminal ends. Modifications are methylations, acetylations, ubiquitinations, SUMOylations, and phosphorylations [66]. Crotonylation, GlcNAcylation, and citrullination are recent histone modifications [67,68]. Chromatin states affect modifications of histone. The methylation of H3K4 facilitates transcriptional activation, the trimethylation of H3K27 facilitates inhibition, and deactivation is facilitated by the acetylation of histones [69]. Metabolic changes in cancer cells are increasingly associated with histone methylation [70]. The EZH2 promotes H3K27me3 to suppress gene transcription [71]. In tumor cells, EZH2 has been found to impact carbohydrate, amino acid, and lipid metabolism. Cancer cells grow more rapidly under the influence of EZH2, which also regulates the Warburg effect. H3K27me3 promotes glycolysis in pancreatic cancers [72], thereby causing it to grow [73,74]. H3K27me3 mediates EZH2’s suppression of adipogenesis [75]. Fig. 2 outlines the principal modification in histone and associated mechanisms, emphasizing their contribution to histone modification and epigenetic regulation.

Metabolic gene transcription is impacted by acetylation and deacetylation. H3K27 acetylation is increased in cells lacking EZH2, and WNT expression is repressed [75]. As a result of EZH2 inhibition, adipocytes accumulate lipids [76]. STK11 (serine/threonine kinase 11) transcription is inhibited by HDAC11 (Histone deacetylase 11), increasing tumor stemness by promoting the glycolysis pathway [77]. A deacetylation of H3K9 by SIRT6 regulates glucose levels [78]. The UTX gene on the X chromosome activates genes associated with brown adipocyte heat production. A process called demethylation and acetylation removes a methyl group from H3K27me3, (PPARG coactivator Alpha and uncoupling protein 1) adds an acetyl group to H3K27 (H3K27ac). UTX plays a significant role in thermogenesis [79]. Recent discoveries have shown that histone Lysine residues on histones are modified epigenetically [11]. p53–P300 and p53–E1A-binding protein interact to mediate this modification. Macrophage M1 division is in its late stages; it promotes gene transcription and the development of M2-like features [80,81]. The SIRT (sirtuin), HDAC (histone deacetylase), and PRMT (protein arginine N-methyltransferase) families of histone modification enzymes regulate non-histone proteins, which regulate metabolic enzymes that are crucial to tumor development. ACETYlation regulates tumor metabolism by the SIRT family of type III deacetylases. IDH2 is inhibited by SIRT3 when it is acetylated at K413 and dimerized, thereby inhibiting glycolysis. DCAF1/DDB1/CUL4B inhibits the degradation of TR4 by SIRT7 [82–84]. ENO2 is an enzyme that is key to glycolysis, and its activity is inhibited when acetylated. Two enzymes regulate this process: at the end of macrophage division, HDAC3 and PCAF are activated [85]. Table 2 summarizes the major histone modification pathways and their mechanisms, with a focus on their roles in epigenetic regulation.

**Table 2:** Mechanisms and pathways of histone modifications with their epigenetic mutations in genes

Pathway for modifying histones	Involved mechanism	Mutation in epigenetics
Methylation of histones [86]	Patterns of dysmethylation, A mutation in H3K27M or overactivity of EZH2 causes H3K27me3 (Histone H3 lysine 27)	EZH2, H3F3A
Histones Acetylation [87]	HATs and HDACs are enzymes that regulate gene expression by acetylating or deacetylating histone proteins.	HDAC2, CREBBP/EP3 00
Ubiquitination of histones [88]	Gene expression regulation and DNA damage response Instability in the genome	Ring1B (RNF2)
Small Ubiquitin- like Modifier (SUMOylation)of histones [89]	Expression and proliferation of tumor suppressor genes. There is less research on brain tumors	UBC9
GlcNAcylation of histones [90]	Serine and threonine residues are supplemented with N- acetylglucosamine (GlcNAc). Epigenetic remodeling and metabolic reprogramming.	OGT
Citrullination of histones [91]	Citrulline is produced by arginine conversion. Transcriptional activation and chromatin decondensation	PADI4
Crotonylation of histones [92]	Expression of genes, There is a lack of understanding of brain tumor	P300rs

Note: Abb: EZH2: Enhancer of Zeste Homolog 2, H3F3A: H3.3 Histone Family Member A (gene encoding histone variant H3.3), HDAC2: Histone Deacetylase 2, CREBBP: CREB-Binding Protein, EP300 (p300): E1A Binding Protein p300, Ring1B (RNF2): Ring Finger Protein 2, UBC9 (UBE2I): Ubiquitin-Conjugating Enzyme E2 I, OGT: O-Linked N-Acetylglucosamine (O-GlcNAc) Transferase, PADI4: Peptidyl Arginine Deiminase 4, P300rs: protein 300 Response Signature.

In glioblastoma, lower overall H3K27me3 levels are strongly associated with poor survival, aggressive mesenchymal transition, and resistance to radiotherapy [93,94]. Conversely, increased H3K4me3 at promoters of glycolytic and stemness-related genes correlates with enhanced tumour-initiating capacity and worse prognosis [95]. Pediatric and young-adult midline gliomas with the H3K27M mutation experience over 80% global reduction of H3K27me3, leading to persistent activation of oncogenic transcriptional programmes; these tumours typically have median survival times under 12 months [96,97]. Schwartzentruber et al. identified the H3K27M histone mutation as a crucial marker in paediatric diffuse midline gliomas, marking a significant advance in understanding CNS tumour biology [96]. This mutation disrupts normal histone methylation by blocking the PRC2 complex, leading to widespread loss of H3K27 trimethylation and broad epigenetic repression. The mutation links tumour metabolism to chromatin regulation because key metabolites, such as α-ketoglutarate (α-KG) and S-adenosylmethionine (SAM), influence methylation processes. These insights show how metabolic alterations in gliomas can drive malignant transformation by directly modifying the epigenetic landscape.

In brain metastases from breast cancer, elevated levels of H3K9 acetylation (H3K9ac) and H3K14ac are linked to increased BBB transmigration and higher expression of adhesion and migration genes; patients with high H3K9ac metastases have significantly shorter progression-free survival [27,98]. Similarly, lung cancer brain metastases show increased H3K36me2 and H3K36me3 levels, which promote transcriptional elongation of metastatic colonisation genes like ST6GALNAC5 and COX2; these heightened H3K36 methylation patterns are associated with earlier intracranial relapse and reduced survival [99].

#### 2.1.3 Chromatin Remodeling and Transcriptional Plasticity

ATP-dependent chromatin remodelers such as SWI/SNF complexes enable dynamic transcriptional adaptation. In the brain, metastatic cells activate neural-mimicry transcriptional programs, including synaptic gene expression, to integrate into the neural niche and exploit neuron-derived factors. Ref. [7] demonstrated that chromatin architecture in the human brain varies widely across cell types, with neurons and glial cells exhibiting distinct 3D genome structures that underpin their specific gene expression patterns. In gliomas and brain metastases, this architecture undergoes significant rewiring, leading to abnormal chromatin loops that either activate oncogenes or silence tumour suppressors by altering enhancer-promoter interactions. These tumour-specific structural changes exemplify a broader epigenetic flexibility, enabling cancer cells to acquire hybrid or stem-like states that enhance malignancy, heterogeneity, and therapy resistance.

#### 2.1.4 Contribution of Non-Coding RNA in Epigenetic Modification

MicroRNAs control cell proliferation and mobility during brain tumor metastasis. Brain metastases caused by lung cancer are associated with miR-328 and miR-378. In brain metastases, microRNAs that suppress metastasis, such as miR-145 and miR-509, are downregulated. KLF4 modulates the stem-like capacity of cancer cells through miR-7. Cancer stem cells have lower levels of miR-7. Metastasis in cancer is negatively affected by improper lncRNA regulation. Tumors found to have metastasized to the brain, such as NSCLC (Non-Small Cell Lung Cancer), are overexpressed by Metastasis-Associated Lung Adenocarcinoma Transcript 1 (MALAT1). Cell migration and growth are promoted by MALAT1 and HOX Transcript Antisense Intergenic RNA (HOTAIR), both of which contribute to NSCLC brain metastasis. Identifying lncRNAs that participate in brain metastasis requires systematic screening approaches [100]. Tumorigenesis may be attributed to abnormal epigenetic modifications resulting from dysregulated expression of long non-coding RNA (lncRNA). In addition to PRC2, EZH2, SUZ12, CoREST, and CRNDE, these lncRNAs alter chromatin methylation profiles, resulting in H3K27me3-repressive chromatin states.

**Figure 3:**
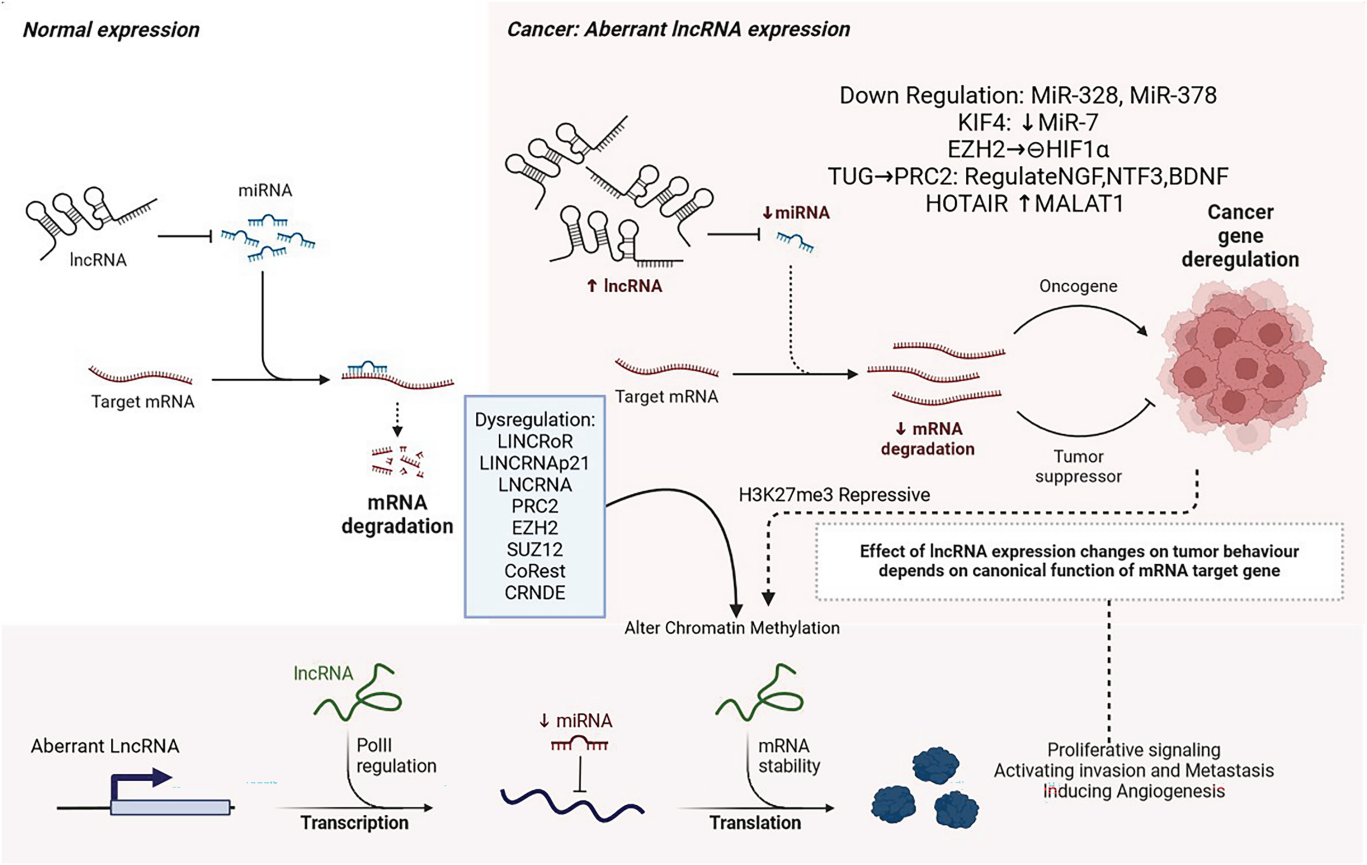
Non-Coding RNA, Created with BioRender.com [103]. In the nuclear chromatin remodeling complex, LncRNA interacts with target loci to regulate their expression. In addition to acting as decoys or guides, LncRNAs regulate transcription through the activity of transcription factors. They are either prevented from binding or promoted from binding to target promoter sequences. LncRNA-protein interactions regulate mRNA stability. As microRNAs compete with lncRNAs for binding to mRNA, lncRNAs modulate mRNA levels. Long noncoding RNAs modulate mRNA translation. LncRNAs induce functional changes in proteins. Abb: long non-coding RNAs (lncRNAs), KIF4 (Kinesin Family Member 4), TUG1 (Taurine Upregulated Gene 1), and CRNDE (Colorectal Neoplasia Differentially Expressed), microRNAs (miRNAs), NGF (Nerve Growth Factor), NTF3 (Neurotrophin-3), BDNF (Brain-Derived Neurotrophic Factor), SUZ12 (Suppressor of Zeste 12), Polycomb Repressive Complex 2, CoREST (REST Corepressor 1)

LSD1/CoREST/REST complexes use HOTAIR as a scaffold [101]. Glioma cells interact with EZH2 to control cell cycle progression. Additionally, TUG1 binds to components of PRC2, which regulate neuronal differentiation-associated genes (NGF, NTF3, and BDNF) [102]. As well as methylating histone H3K27, YY1 promotes locus-specific methylation. In both normal development and disease, several small nucleic acids modulate DNA methylation. Gliomas exhibit dysregulation of LincRoR and TUNA (Tcl1 Upstream Neuron-Associated lncRNA). Fig. 3 illustrates the epigenetic regulatory mechanisms mediated by non-coding RNAs.

LincRNAp21 supports the promoters of pluripotency genes methylated at CpG sites to prevent somatic cell reprogramming. Clinical trials are incorporating lncRNA data in their research on glioma. A link between abnormal expressions of long noncoding RNAs in gliomas and glioblastoma (GBM) prognosis has been identified [101]. Studies suggest that non-coding RNAs regulate sugar, lipid, and cholesterol metabolism in tumors [102,104]. Research on the role of noncoding RNAs in tumor metabolism is still limited. Furthermore, lactic acid production is stimulated as well as glucose uptake. Urothelial Cancer Associated 1 (UCA1) increases metastasis by activating HK2. Inhibition of miR203 can suppress glucose metabolism [105]. In cervical cancer, CircCDKN2B-AS1 binds to IMP U3 small nucleolar ribonucleoprotein 3 (IMP3) as a cyclic structure, stabilizes transcription, and promotes aerobic glycolysis (HK2) [71]. HIF1α regulates tumor glucose metabolism by upregulating the transcription of enzymes and transporters encoding glucose. Long noncoding RNAs regulate the HIF1α pathway. The lncRNA-p21 binds to HIF1α, inhibits LncRNA-p21 binds to HIF1α, inhibiting its ubiquitination and promoting glycolysis in hypoxia, influencing the Warburg effect [106,107]. HIFAL A non-coding RNA inhibiting glycolysis, binds to PKM2, allowing PHD3 to hydroxylate PKM2’s proline residue, and then integrates PKM2/PHD3 into the nucleus [108]. Meanwhile, HISLA prevents HIF1α degradation by inhibiting the interaction between PHD2 and HIF1α. This leads to increased glycolysis and inhibition of programmed cell death [109]. The transcription of many glucose metabolism genes is initiated by HIF1α [110]. Tumor development relies heavily on miRNAs. Nasopharyngeal carcinoma cells exhibit metabolic reprogramming induced by miR-9-1, while GBM cells manifest glycolysis induced by miR-542-3p [104,111]. As a result of the inhibition of the Golgi phosphoprotein 3 (GOLPH3L) by miRNA-1185-2-3p, the GOLPH3L gene regulates the central carbon metabolism, which is altered [112]. A study found that LIX1L (Limb and CNS Expressed 1-Like) increased miR-21-3p and inhibited FBP1 (Fructose-1,6-Bisphosphatase 1), which resulted in lactic acid reduction, sugar metabolism manipulation, and tumor growth inhibition. Inhibition of fatty acid synthesis suppresses the growth of lung cancer cells. MiR-15a-5p inhibits acetate uptake under hypoxia, as well as ACSS2 and H4 acetylation [113,114].

## 3 Metabolic Reprogramming in Cancer

As a source of energy, brain tumor cells need glucose. In addition to increased biosynthesis and energy production, cancer cells need metabolic reprogramming [115]. There is minimal genetic impact from cancer metabolism [116]. A cancer cell’s epigenetic state is closely related to its metabolic state. When cells are reprogrammed, cofactors for epigenetic enzymes can be altered. A cancer cell’s epigenetic profile can be altered after these changes affect enzymatic activity. A key metabolic pathway can be controlled by epigenetic changes, which can also affect the expression of key enzymes [117]. To fuel and maintain their proliferation and tumor growth, cancer cells are known to undergo metabolic reprogramming. Metabolic reprogramming involves altering several energy pathways.

### 3.1 Key Features of Metabolic Reprogramming

#### 3.1.1 Warburg Effect and beyond

Often, aerobic conditions favour glycolysis over oxidative phosphorylation. (Warburg effect) [118]. Metastatic brain tumors rely on more complex metabolic strategies than the traditional Warburg effect alone. They use metabolic symbiosis, where astrocytes or stromal cells export lactate via Monocarboxylate Transporter 4 (MCT4), and tumor cells import and oxidize it through Monocarboxylate Transporter 1 (MCT1) to support mitochondrial metabolism and redox balance. This lactate shuttle also fuels epigenetic changes such as histone lactylation. In addition, immune-metabolism pathways including the tryptophan IDO1/kynurenine axis and macrophage-mediated arginine depletion create a profoundly immunosuppressive tumor microenvironment while also influencing chromatin regulation [119,120]. Is there a similar metabolic adaptation for all tumor cells, or does each subgroup require a different strategy? One study found that brain tumor-initiating cells (BTICs) are less lactate-producing and glucose-consuming than differentiated stem cells. BTICs have higher mitochondrial reserves and are more radioresistant than differentiated tumor cells. Past research suggested that BTICs are exclusively oxidatively phosphorylated [115,121]. Cancer stem cells (CSCs) are capable of coping with oxidative stress and avoiding redox imbalances because they undergo a metabolic deviation from glycolysis to PPP. Hu et al. discuss, histone lactylation, a novel post-translational modification caused by elevated lactate levels, enhances gene expression in hypoxic tumour microenvironments [122] This feature is characteristic of glioblastoma (GBM) and brain metastases. The process links altered tumour metabolism with transcriptional activation, enabling cancer cells to adapt and survive under low oxygen conditions. Their findings demonstrate how metabolic byproducts like lactate can directly influence chromatin states, promoting malignancy by sustaining gene expression in aggressive brain tumors. Although it is a poor clinical biomarker due to low specificity, spatial heterogeneity, and confounding by non-tumor conditions such as ischemia or seizures [123].

MB stem cells might be targeted with inhibitors of G6PD and 6GPD combined with ROS-inducing therapies. *In vivo* and *in vitro* G6PD inhibitors can confer resistance in different cancer types by activating alternative metabolic pathways to PPP. CSCs with a PPP-dependent phenotype for killing can be selected using NRF2, the master regulator of antioxidant defense [124]. Children’s brain tumors, including NRF2, are resistant to therapy. *In vitro*, targeting NRF2 has synergistic antitumor effects on MB, whereas NRF2 expression is correlated with adult GBM. Hypoxic, acidic, and nutrient-deficient tumor microenvironments (TMEs) are found in MB. Cell differentiation in tumors is blocked by hypoxia, caused by uncontrolled cancer cell proliferation. HIF1α regulates therapy resistance. Autophagy metabolism and TME nutrients [124]. It is essential for cancer cell proliferation that glutamine fuels the TCA cycle. When T-cells are activated, the MAPK/ERK-pathway regulates glutamine uptake and glutaminolysis, which can negatively impact T cell and antitumor immunity [125]. Glutamine blockade may have an indirect immunostimulatory antitumor effect by inhibiting MDSC recruitment and generation via CSF3 inhibition. Glutamate deprivation induces PD-L129 expression [126].Through the kynurenine pathway, cells utilize tryptophan as an essential amino acid. Type I and type II interferons induce tryptophan catabolism, a mechanism for inhibiting antitumor immunity [127]. CD8^+^ T cells are inhibited by tryptophan starvation, while CD4^+^ regulatory T (Treg) cells are stimulated, creating a tolerogenic tumor microenvironment by inhibiting immune checkpoints such as Pathways involving CTLA4 and PD1/PD-L1 [128]. Metabolites generated from tryptophan catabolism suppress T cell immunity. Kynurenine (metabolite product, IDO-dependent pathway of tryptophan degradation) is exported by cancer cells into the tumor microenvironment in order to inhibit antitumor immunity and tumor clearance [128,129]. The combined effects of tryptophan depletion and kynurenine-mediated AhR translocation have already been shown to facilitate tumorigenesis by inducing regulatory T cell phenotypes [128,130]. A limited amount of arginine is available in tumor microenvironments. The absence of arginine in immune cells interferes with glycolysis, inhibiting T-cell proliferation and cytokine production. Cancer cells often starve for arginine as a second line of defence [131,132].

#### 3.1.2 Cancer Stem Cell Metabolism

A three-model approach is applied to discuss how metabolic regulation affects epigenetic regulation in cancer cells. TETs and KDMs are inhibited by inhibitory metabolites (e.g., α-KG), leading to increased aberrant histone and DNA methylation. Model 1, which suggests metabolite-mediated inhibition of epigenetic enzymes, aligns closely with evidence that TET and KDM dioxygenases are hindered by reduced α-KG or increased inhibitory metabolites such as 2-HG, fumarate, and succinate [133]. The classic example of IDH-mutant gliomas showing global DNA and histone hypermethylation (G-CIMP) due to 2-HG–mediated TET inhibition. Extending this model to brain metastasis is straightforward, as these tumors also experience α-KG scarcity caused by glutamine dependence and hypoxia. Model 2, which focuses on nutrient-sensing pathways, is supported by ample evidence that HIF1α-driven glycolysis and lactate build-up reshape chromatin, including the induction of histone lactylation in hypoxic GBM and metastases. Incorporating these HIF1α-dependent epigenetic changes would create a direct mechanistic link between nutrient sensing and chromatin adaptation. Model 3, emphasizing metabolite production as a direct substrate for epigenetic modification [134], is supported by studies showing that acetyl-CoA availability controls H3/H4 acetylation, while lactate fuels histone lactylation all elevated in brain tumors that rely on acetate, lactate shuttles, and mitochondrial respiration during nutrient limitation. Clearly linking each model to these experimental findings would shift the framework from purely conceptual to mechanistically grounded, making it more convincing for readers and more valuable for identifying therapeutic vulnerabilities in metastatic brain tumors.

Key metabolites involved in the crosstalk between cancer metabolism and epigenomic regulation are summarized in Table 3.

**Table 3:** Key metabolites in cancer metabolism interplay between epigenomics and metabolism in cancer

Metabolite	Pathway	Role in metabolism	Effect on epigenetics	Key points	Clinical trial ID
Glucose ([135,136] Izzo & Wellen, 2019; Lee et al., 2014)	Glycolysis	Adenine dinucleotide and pyruvate are produced as glucose/glycogen is broken down	Under anoxic conditions, pyruvate is reduced to lactate, which can be involved in histone lactylation	Lactate production links metabolism with gene regulation through lactylation	NCT06059690
Acetyl-CoA [136]	Glucose, amino acid, and fatty acid metabolism	Formed from glucose, amino acids, and fatty acids metabolism; substrate for acetylation	Regulates histone and non- histone protein acetylation; implicated in cancer development	Acetylation is crucial for tumorigenesis and development. Tumor proliferation is linked to acetyl- CoA generation via ACLY and ACSS1	NA
NAD^+^(Nicotinami de Adenine Dinucleotide) [137,138]	Sirtuin activity	A sirtuin and class III HDACcofactor that deacetylates lysine	NAD^+^/NADH ratio alteration is associated with diseases like cancer; affects sirtuins activity	High NAD^+^ levels in cancer cells reprogram metabolism to support tumor progression	NCT03514875
TCA Intermediates (α-KG, Succinyl-CoA,Fumarate) [139,140]	TCA cycle intermediate	Involved in mitochondria l function and energy production	Regulate epigenetic modifications; mutations in TCA cycle enzymes can lead to cancer	α-KG affects histone demethylation. Succinyl-CoA and fumarate mutations lead to epigenetic reprogrammingand cancer	NCT02704858; NCT00741403
2-Hydroxyglutarate (2-HG) [53,135]	Isocitrate dehydrogenase mutations	Oncometabol ite produced by mutant IDH; affects methylation and demethylation	Linked with global hypermethylatio n in cancers like AML; affects prognosis	D-2HG and L- 2HG have roles in epigenetic modifications and tumor progression	NCT01703962
Lactate [141]	Pyruvate metabolism	By-product of anaerobic glycolysis; substrate for histone lactylation	Histone lactylation linked to gene transcription and tumorigenesis	Indicates poor prognosis in cancers like ocular melanoma	NCT07173829; NCT07211841
Lipid metabolism [142,143]	Palmitic Acid-Fatty acid metabolism, Farnesyl Group- Protein prenylation Geranylgeranyl Group Protein	Produces substrates like acetyl- CoA for energy metabolism and epigenetic modifications	Lipids modify proteins affecting cancer cell proliferation and immune evasion	Palmitoylation, Farnesylation, Geranylgeranylati on, and β- Hydroxybutyrate modifications are crucial for tumor progression	NCT00612651; NCT00038493; NCT00050986

Note: Abb: tricarboxylic acid (TCA), ATP Citrate Lyase (ACLY), Acyl-CoA Synthetase Short-Chain Family Member 1 (ACSS1), Alpha-Ketoglutarate (α-KG).

Epigenetic landscapes are differentially affected by tumor cells’ metabolite sensors [144]. A proliferative profile is favored by metabolic cooperation among tumor cells [145,146]. As chromatin modulators alter and epigenetic marks reorganize, cell metabolism can facilitate cell transitions. Metabolic reprogramming can occur when nutrients are available in the tumor microenvironment. As a result of metabolic reprogramming induced by hypoxia, gene expression can be reprogrammed and chromatin remodelled, leading to the creation of cells with new phenotypes [116]. Cells die from synthetic lethality when two genes interact. Synthetic lethality offers an underexplored therapeutic approach in brain metastasis, where BBB/BTB-driven metabolic constraints create vulnerabilities not found in extracranial tumours. Brain-colonising cancer cells rely heavily on glutamine to sustain the TCA cycle, maintain α-KG levels, and support α-KG–dependent epigenetic enzymes such as TETs and KDMs; thus, glutamine deprivation leads to H3K27 hypermethylation and diminishes the effectiveness of targeted agents like BRAF inhibitors. In the hypoxic, acidic, lactate-rich environment of the brain microenvironment, this metabolic–epigenetic rigidity becomes a powerful synthetic lethal target. Combining glutaminase inhibitors (e.g., CB-839) with EZH2, HDAC, or BET inhibitors may selectively disrupt these compensatory pathways, while co-targeting lactate transporters (MCT1/4) and lactylation-related writers (p300/CBP) addresses vulnerabilities created by tumour–astrocyte lactate shuttling. The manuscript also highlights precedents such as TET3-deficient tumours, which are sensitised to glycolytic blockade (2-DG), and BCAT1 inhibition working synergistically with PARP inhibitors, both of which can be applied to brain metastasis, where metabolic bottlenecks are further intensified.

Cancer patients with epigenetic or metabolic deficiencies can benefit from BCAT1 inhibitors if they have BRCA mutations or PARP inhibitions. In LUAD cells with KMT2D loss, PER2 no longer inhibits glycolytic gene expression, providing an attractive therapeutic vulnerability. The 2-DG inhibitor inhibits glycolysis in AML cells deficient in TET3, and this inhibits sensitive to 2-DG in xenotransplantation models [147]. Epigenetic agents can be vulnerable to metabolic deficiencies. Lack of glutamine, for instance, leads to hypermethylation of histone H3K27, which reduces the effectiveness of BRAF inhibitors in treating melanoma cells. New-generation epigenetic drugs may improve the efficacy and tolerability of synthetic lethal therapies [45]. The tumorigenic potential is maintained by manipulating cellular metabolism and epigenetics. Fission or fusion is possible within mitochondria. RTG signaling pathways connect them to the nucleus. An RTG response can result from metabolic deficiencies. Gene transcription depends on TET enzymes, ACoA, NNMT, and NAD^+^ metabolism. Demethylation of histone H3K4 residues is carried out by KDM1 using FAD [148]. Glycine N-methyltransferase creates a metabolic methylation sink in cancer. Small-molecule inhibitors can reverse epigenetic dysfunction caused by IDH2 and TET2 mutations in acute myeloid leukemia. Through newly acquired mutations in IDH, cancer cells can become resistant to IDH inhibitors [149]. In PARP inhibition, nuclear PKM2 must be targeted. Brain tumors, as well as acute myeloid leukemia, can be treated with new IDH1 mutant inhibitors. A-KGDH stimulates histone succinylation at tumor-promoting genes through histone H3 succinyltransferase. Lysosomal biogenesis and autophagy genes are induced by ACSS2 in cancer cells [150]. Nuclear acetyl-CoA synthetase 2 (ACLY) facilitates DNA repair through homologous recombination by increasing histone acetylation at double-strand breaks [151]. By activating proton-sensing GPR4 and GPR68, acidosis activates a family of GPCRs that may regulate tumor cell function and metabolism [152]. Through inhibition of glycolysis, acidosis increases the TCA cycle and activates p53 in tumor cells. As P53 regulates Parkin, a Parkinson’s disease-associated gene, oxidative phosphorylation is enhanced, and glycolysis is reduced. P53 also modulates ROS production [153]. AKT, c-Myc, and HMGB-1 are suppressed by acidosis, as well as the expression of several proto-oncogenes [154]. Metabolic shifting is dependent on MEOX2 in hypoxia and glycolysis. A knockdown of MEOX2 inhibits the expression of four metabolic genes [155].

### 3.2 Therapeutic Targeting of Metabolic Reprogramming

**Figure 4:**
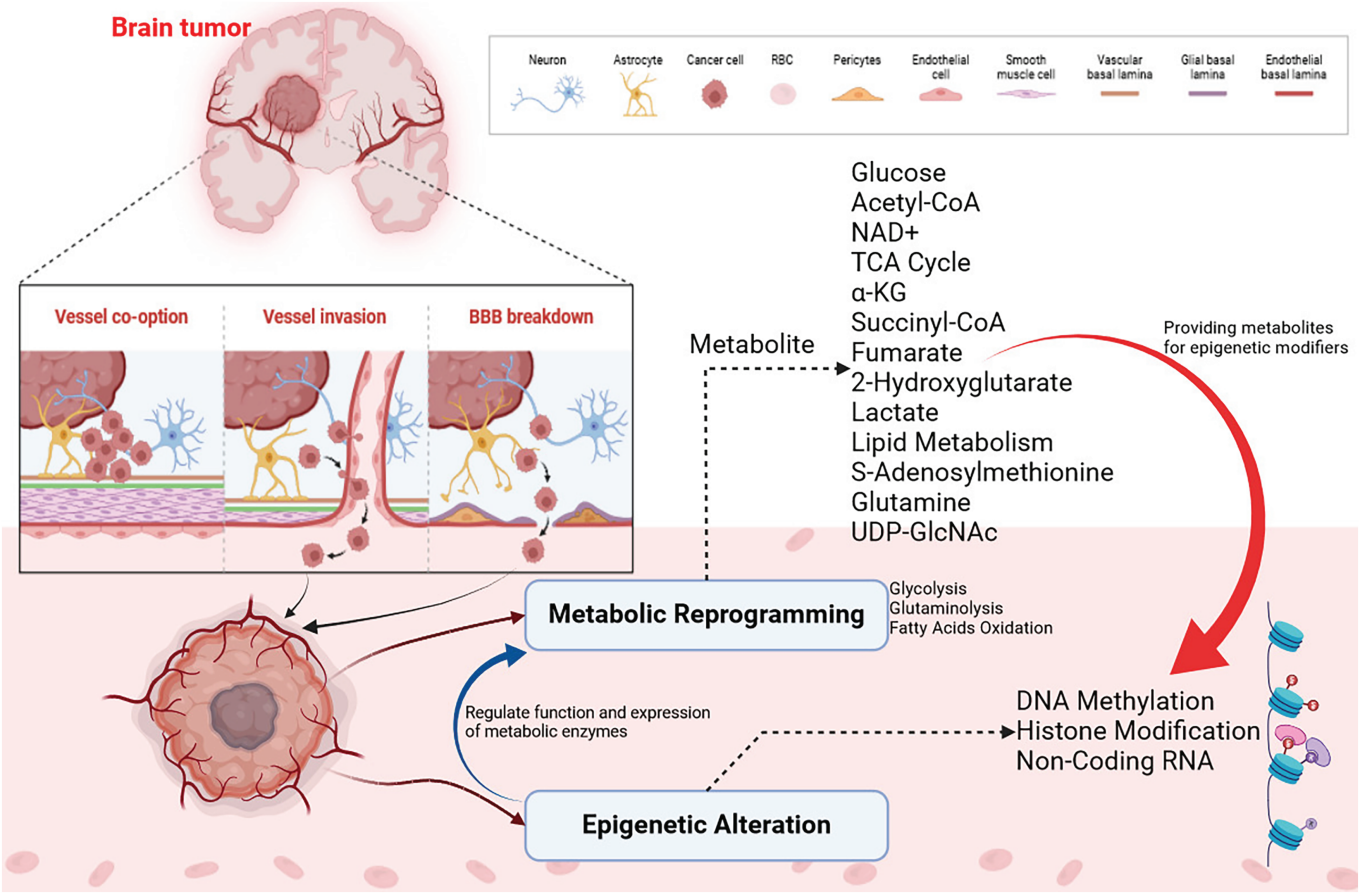
Metabolic–epigenetic pathway map illustrating how key metabolites regulate chromatin remodeling in metastatic brain tumors. Tumor cells infiltrate the brain microenvironment via vessel co-option, direct vessel invasion, and blood brain barrier (BBB) disruption. The cancer cells interact with neurons, astrocytes, endothelial cells, pericytes, and smooth muscle cells, reshaping the vascular basal lamina. Metabolic reprogramming is characterized by enhanced glycolysis, fatty acid oxidation, and TCA cycle remodeling, generating key metabolites such as glucose, acetyl-CoA, NAD^+^, α-ketoglutarate (α-KG), succinyl-CoA, fumarate S-adenosylmethionine (SAM) and others which serve as cofactors and substrates for epigenetic enzymes, influencing DNA methylation, histone modifications and noncoding RNA expression. These epigenetic alterations modulate chromatin accessibility, transcriptional plasticity, and immune evasion, enabling tumor adaptation and therapeutic resistance in the brain. Created through BioRender.com [167]

Could these patients benefit from targeted metabolic therapeutics? There have been promising preclinical data on glycolytic and mitochondrial therapeutics. It is well known that 2-deoxy-D-glucose causes toxicity at high doses due to its ability to target all hexokinases [156,157]. There is, however, strong evidence for the potential reduction in toxicity from developing a selective HK2 inhibitor. Preclinical animal studies indicate that PKM2 activators are promising immunotherapies for cancer. There has been evidence that YM155 inhibits HK2 *in vitro* and *in-vivo* models, and how glucose oxidation replaces glycolysis in metabolism [158]. High-grade gliomas have higher amino acid, lactate, glycerophosphocholine, and lactate levels when analyzed metabolomically with nuclear magnetic resonance [159]. Lactate acts as a biomarker for several diseases as an indicator of poor tissue perfusion. Researchers are unclear about the role of elevated serum lactate in brain tumours. The highest serum lactate levels are found in GB, establishing a noninvasive biomarker for brain tumor grade [157]. Oncogenic phenotypes are seen because of tumor metabolism rather than just an indirect consequence of tumor growth in cancer metabolism research. A significant component of GB treatment will be testing therapeutic targets to inhibit aberrant glucose metabolism, redefining these ideas, and developing preclinical models. With this approach, cancer treatments could be more precise and personalized, resulting in a revolution in cancer treatment. Also, metastasis and recurrence risks could be reduced [121]. The interaction between HIF1α and Neurogenic locus notch homolog protein (NOTCH) signaling enhances cell stemness in MB cells. Targeting the HIF1α-NOTCH axis could benefit high-risk MB patients. HIF1α and NOTCH signaling have been shown to work together to promote cell proliferation, survival, and differentiation. Targeting the pathway could inhibit the growth and survival of MB cells, thereby improving outcomes for high-risk patients. GLUT1, PDK1, and CAIX can be targeted for hypoxic and metabolically adapted MB [160]. Optimal and synchronized energy use with growth may be achieved in complex organisms via the epigenomic-metabolic axis; there may be a link between metabolic regulation and acetylation through circadian rhythm and peripheral clocks [160,161]. Hearts and muscles, among other tissues, are exposed to starvation, which reduces acetyl-CoA levels and protein acetylation. ACETYL-CoA production is promoted by strategies that activate PDCs, such as dichloroacetate (DCA), which promotes acetyl-CoA production [162]. In contrast, starvation increases liver and protein acetyl-CoA levels but not brain acetyl-CoA levels [163]. Circulating ketone bodies, such as those produced by the liver during starvation, may regulate acetylation mechanisms in other organs, thereby explaining these organ-specific effects. Beta-hydroxybutyrate inhibits lysine deacetylases (KDACs) [164]. The other important thing to remember is that different parts of the body have distinct sources of acetyl-CoA, such as neurons, which produce it from beta-hydroxybutyrate, and hepatocytes, which generate it from ethanol [165,166]. The data demonstrates how context, cell type, or organ can influence mechanisms of acetyl-CoA homeostasis. In studying cancer across organs, we must consider the metabolism-epigenetic axis. Dysregulation of metabolic homeostasis may play a role in cancer cachexia. An AMP-activated protein kinase (AMPK) and acetyl-CoA metabolic disturbance have been linked to cancer cachexia muscle wasting, which may be a therapeutic target. For example, muscle wasting associated with cancer cachexia has been reduced in mice with AMPK activation [146]. An integrated metabolic–epigenetic framework underlying chromatin remodeling and tumor adaptation in brain metastases is shown in Fig. 4.

### 3.3 Metabolism Reprogramming: Strategies for Reversal

**Figure 5:**
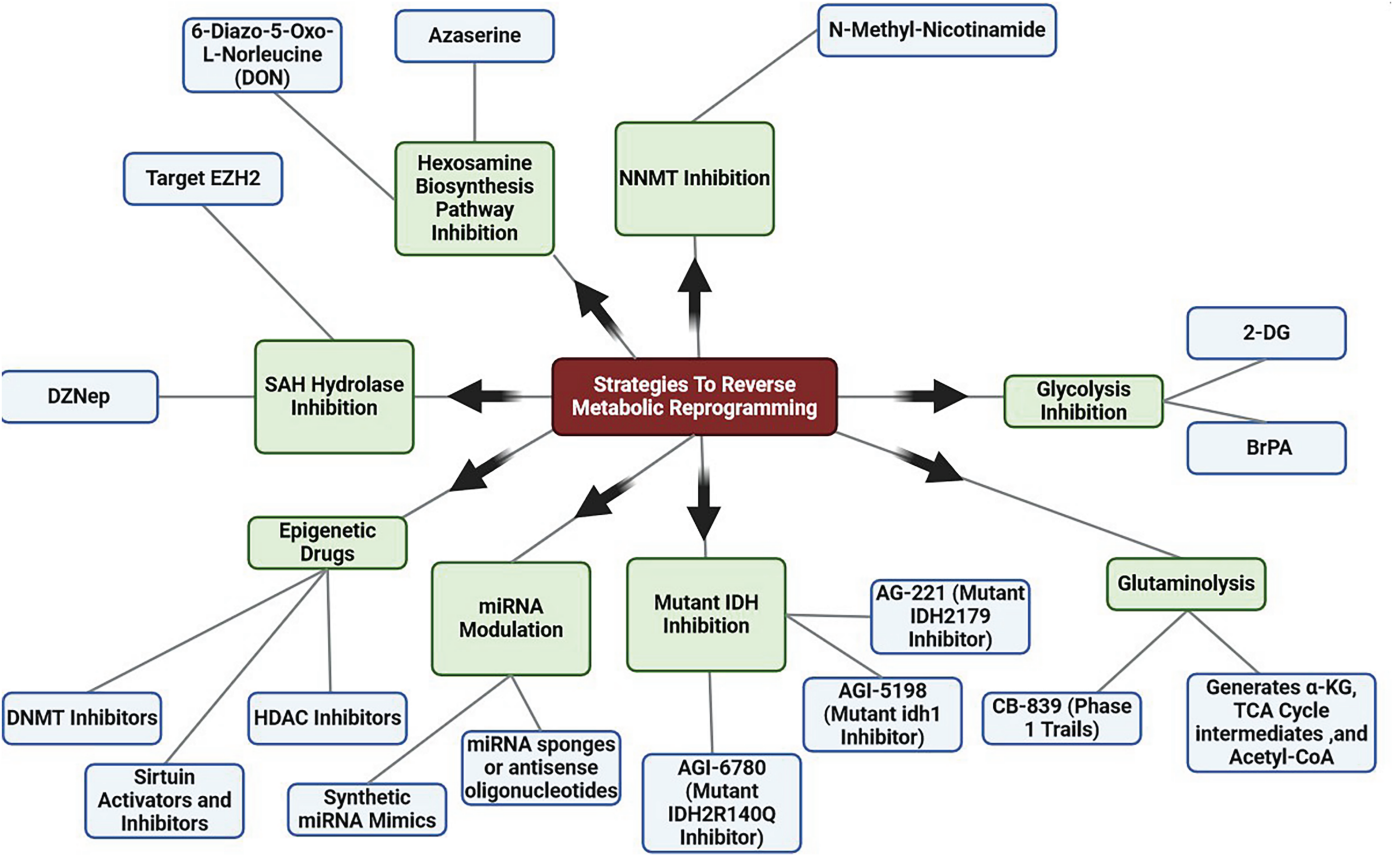
Overview of strategies to reverse metabolic reprogramming. Therapeutic strategies to reverse metabolic reprogramming include targeting glycolysis, glutaminolysis, mutant IDH enzymes, SAH hydrolase, and hexosamine biosynthesis, alongside miRNA modulation and epigenetic drugs which aim to restore metabolic balance and suppress tumor-promoting transcriptional programs Created with BioRender.com. Abb DZNep, (3-Deazaneplanocin A), SAH (S-Adenosyl-L-Homocysteine), BrPA (3-Bromopyruvate), 2-DG (2-Deoxy-D-Glucose)

An antitumor response against MB is induced by pharmacological targeting of NRF2. In cancer, acetyl CoA and citrate are produced at a faster rate, which contributes to histone acetylation. DNA repair and cell sensitization to DNA-damaging therapies may be possible by inhibiting glycolysis (e.g., 2-DG or 3-Bromopyruvate; BrPA) [168] (Moussaieff et al., 2015). Several inhibitors of glutaminolysis have been identified, a process frequently elevated in cancer. Currently, CB-839 is under testing for solid and hematological malignancies in Phase I dose escalation trials [169] (Robinson et al., 2007). The TCA cycle, α-KG intermediates and acetyl-CoA are among the epigenetic factors formed during glutaminolysis. Zaprinast is an unbiased small molecule that binds to the IDH1R132C enzyme and blocks its activity. This inhibition prevents the enzyme from producing 2-HG, the primary metabolite associated with the mutant IDH1R132C and known to cause cancer [170] (Elhammali et al., 2014). The goal of suppressing oncometabolite 2-HG production is achieved by utilizing IDH1/2 inhibitors. IDH1, R132H, was the first selective inhibitor of mutant IDH1, AGI-5198 appears to be an effective tool for selecting IDH1 over wild-type IDH1. AG-221 selectively inhibits IDH2179 mutants, while AGI-6780 selectively inhibits IDH2R140Q mutants. In cancers bearing mutant forms of IDH1/IDH2, targeting mutant forms of these proteins may offer clinical benefits as differentiation therapy [171] (Kernytsky et al., 2015). Hydrolases that convert S- adenosylhomocysteine to adenosine and homocysteine are essential for maintaining homeostasis in the methylation process. DNA and histone methylation are inhibited by DZNep, an inhibitor of SAH hydrolase [172] (Miranda et al., 2009). DZNep suppresses the expression of numerous developmental genes by inhibiting SAH hydrolase and reactivating EZH2, an oncogenic HMT, but it is less effective against genes that have dense promoter methylation [173] (Momparler & Côté, 2015). An inhibitor of NNMT, N-methyl nicotinamide, is a reaction byproduct. As a result, methylated H3K4 binds more strongly to gene promoters, and histone methylation at H3K4 increases [174] (Kraus et al., 2014). Glucose drives the proliferation of cells, but two inhibitors of the hexosamine biosynthesis pathway reduce the expression of catenin and inhibit protein O- GlcNAcylation. The inhibitors are 5-diazo-5-oxo-L-norleucine (DON) and O-diazoacytyl-L-serine (azaserine) [175,176] (Olivier-Van Stichelen et al., 2012; Zhou et al., 2016). A complementary approach to counteracting oncometabolites in cancer may be to modulate cancer metabolism with epigenetic drugs. Myelodysplastic syndrome and acute myeloid leukemia are two diseases that can be treated with DNMT inhibitors. They are nonspecific cytosine analogs that inactivate DNMT1, DNMT3A, and DNMT3B [177] (Rodríguez-Paredes & Esteller, 2011). Cancer metabolism may be affected by HDAC inhibitors, which induce histone acetylation and reverse gene silencing. Among the approved cancer treatments, butyrate and trichostatin A are HDAC inhibitors [178] (Alcarraz-Vizán et al., 2010). Sirtuin activators and inhibitors regulate cancer metabolism. Activators and inhibitors of sirtuin are needed to determine their effects on cancer metabolism and their efficacy against cancer [179] (Villalba & Alcaín, 2012). Considering their regulatory functions in metabolic dysregulation, miRNAs are promising therapeutic targets in cancer. Currently, miRNAs can be targeted in two ways. A synthetic miRNA mimic can restore aberrantly silenced miRNAs, while antisense oligonucleotides or miRNA sponges can silence overexpressed miRNAs [180,181] (Bader et al., 2010; Li & Rana, 2014). Metabolic gene manipulation in cancer has been achieved using both approaches. An example is miR-143, which targets the 3^′^-untranslated region of hexokinase II, thereby inhibiting cell growth and reversing glycolytic phenotypes [182] (Gregersen et al., 2012). Fig. 5 illustrates therapeutic approaches designed to counteract metabolic reprogramming in cancer cells.

## 4 Limitations and Future Perspectives

There is a strong and reciprocal relationship between metabolism and epigenetics, and this crosstalk plays a central role in cancer biology. Metabolically dependent epigenetic modification is explored to identify novel cancer targets. However, cancer remains incompletely understood in large part due to the complexity of the metabolic–epigenetic axis. A constantly changing environment is influenced by altered metabolism and epigenetic deregulation. It is imperative to understand how metabolites regulate epigenetic targets. Epigenetic modifications link metabolic pathways and gene expression. Cancer metabolism and epigenetics have made significant advances in recent years, as highlighted in this review. Carcinogenesis and metastasis mechanisms are clarified by it. This article emphasizes the importance of taking a multifaceted approach to fully comprehend the complexity of cancer biology, emphasizing the potential for targeting this crosstalk to develop effective cancer prevention and therapy strategies. A tumor’s epigenetic landscape is altered because of metabolic reprogramming. Several biochemical enzymes can modify chromatin by acting upon metabolites that act as epigenetic substrates. Consequently, future studies on cancer biomarkers and therapeutic strategies must account for the metabolism epigenome axis. Despite extensive research, many questions remain unanswered: how exactly do metabolic and epigenetic interactions drive tumorigenesis, how can these processes be prevented, and how can they be effectively targeted? A deeper biochemical understanding of the epigenomic landscape is essential. Much of the current knowledge is derived from *in vitro* or non-brain-specific models, leaving uncertainty about how these mechanisms operate within the highly specialized and heterogeneous brain microenvironment. Disentangling tumor cell–intrinsic metabolic programs from those imposed or supported by astrocytes, microglia, and endothelial cells remains a major challenge. Therapeutically, selectively targeting metabolic–epigenetic pathways in tumors without disrupting essential brain functions remains a significant obstacle. Overcoming these gaps will require advanced multi-omics technologies, improved *in vivo* models, and careful therapeutic design to achieve both specificity and efficacy. The integration of multi-omics platforms, particularly metabolomics with epigenomics, aims to identify robust biomarkers of brain metastasis progression and treatment response. Incorporating spatial transcriptomic and epigenomic technologies will be vital for resolving metabolic and chromatin heterogeneity within the brain tumor microenvironment. Finally, leveraging metabolic–epigenetic crosstalk to stratify patients and guide clinical trial design presents a promising translational pathway that could translate these mechanistic insights into precision therapies. Continued research will be key to developing innovative therapeutic applications that exploit this intricate interplay, ultimately advancing cancer prevention and treatment.

## Data Availability

Not applicable.
